# New Insight on the Immune Modulation and Physical Barrier Protection Caused by Vitamin A in Fish Gills Infected With *Flavobacterium columnare*

**DOI:** 10.3389/fimmu.2022.833455

**Published:** 2022-03-25

**Authors:** Wei-Dan Jiang, Li Zhang, Lin Feng, Pei Wu, Yang Liu, Sheng-Yao Kuang, Shu-Wei Li, Ling Tang, Hai-Feng Mi, Lu Zhang, Xiao-Qiu Zhou

**Affiliations:** ^1^ Animal Nutrition Institute, Sichuan Agricultural University, Chengdu, China; ^2^ Fish Nutrition and Safety Production University Key Laboratory of Sichuan Province, Sichuan Agricultural University, Chengdu, China; ^3^ Key Laboratory of Animal Disease-Resistance Nutrition, Ministry of Education, Ministry of Agriculture and Rural Affairs, Key Laboratory of Sichuan Province, Sichuan, China; ^4^ Animal Nutrition Institute, Sichuan Academy of Animal Science, Sichuan Animtech Feed Co. Ltd., Chengdu, China; ^5^ Tongwei Co., Ltd., Healthy Aquaculture Key Laboratory of Sichuan Province, Chengdu, China

**Keywords:** gill, immune, vitamin A, antioxidant capacity, apoptosis

## Abstract

In this study, we have investigated the influence of vitamin A on gill barrier function of grass carp (*Ctenopharyngodon idella*) infected with *Flavobacterium columnare.* The fish were fed different concentrations of vitamin A diets for 10 weeks and then infected with *F. columnare* by immersion. We observed that optimal vitamin A significantly prevented gill rot morbidity in fish infected with *F. columnare*. Further investigations revealed that vitamin A boosted the gill immunity by increasing the contents of complements (C3 and C4), activities of acid phosphatase (ACP) and lysozyme, mRNAs of *β-defensin-1, liver-expressed antimicrobial peptide 2A* and *2B (LEAP-2A* and *LEAP-2B)*, *hepcidin*, and anti-inflammatory cytokines like *transforming growth factor β1 (TGF-β1), TGF-β2, interleukin-10 (IL-10),* and *IL-11*. It also enhanced the levels of various related signaling molecules including *inhibitor protein κBα (IκBα), target of rapamycin (TOR),* and *ribosome protein S6 kinase 1 (S6K1)* but downregulated the expression of pro-inflammatory cytokines including *IL-1β, IL-8, tumor necrosis factor α (TNF-α)*, and *interferon γ2 (IFN-γ2)* and related signaling molecules including *nuclear factor κB p65 (NF-κB p65)* (rather than *NF-κB p52), IκB kinase β (IKKβ), IKKγ* (rather than *IKKα), eIF4E-binding protein 1 (4E-BP1),* and *4E-BP2* mRNA levels in fish gills. In addition, dietary vitamin A markedly lowered the concentrations of reactive oxygen species (ROS), malondialdehyde (MDA), and protein carbonyl (PC), increased both the activities and mRNAs of copper/zinc superoxide dismutase (Cu/ZnSOD), MnSOD, glutathione transferases (GSTs), glutathione peroxidase (GPx), and glutathione reductase (GR) associated with upregulation of *NF-E2-related factor 2 (Nrf2)* mRNAs and downregulation of *Kelch-like-ECH-associated protein (Keap1a)* and *Keap1b* mRNAs. Moreover, vitamin A decreased the mRNAs of different apoptotic mediators [*caspases 8, 9, 3* (rather than 7)] associated with downregulation of signaling molecule *p38 mitogen-activated protein kinase (p38MAPK)* mRNAs in fish gills. Besides, vitamin A promoted tight junction (TJ) complex mRNAs [including *claudin-b, -c, -3, -7, -12, occludin*, and *zonula occludens-1 (ZO-1)*] that have been linked to the downregulation of myosin light chain kinase (MLCK) signaling. Taken together, the current study demonstrated for the first time that vitamin A markedly enhanced gill health associated with immune modulation and physical barrier protection. Based on protecting fish against gill rot morbidity, ACP activity, and against lipid peroxidation, optimum vitamin A concentrations in on-growing grass carp (262–997 g) were found to be 1,991, 2,188, and 2,934 IU/kg diet, respectively.

## 1 Introduction

Aquaculture is one of the rapidly growing food production sectors, providing almost 50% of fishery products for human consumption (1). However, aquaculture diseases are frequently encountered due to culture intensification, which can cause significant loss of production and hamper the development of aquaculture (2, 3). Gill rot disease is a common bacterial infectious fish disease, which can result in high mortality and heavy economic losses in the aquaculture industry (4). *Flavobacterium columnare* is a major pathogen found in freshwater culture, which can contribute to the high incidence of gill rot disease in fish (5). Thus, it is very important to develop effective approaches for prevention of gill rot diseases in aquaculture. Vitamin nutritional strength strategies have been reported to be efficacious in the prevention of gill rot disease (6). Our previous study has demonstrated that vitamin nutrition, such as vitamin C, could directly reach fish gills and enhance gill immunity as well as decrease the gill rot morbidity by about 75% in grass carp (*Ctenopharyngodon idella*) caused by *F. columnare* infection (7). Vitamin A not only serves as an important nutrient for fish growth but also can exhibit immunoregulatory effects that have been well documented in the animals (8). For example, a previous study from our laboratory showed that vitamin A can significantly enhance the immunity in the intestine and the growth of grass carp (9). However, no prior reports have investigated the potential influence of vitamin A on the immunity in fish gills. It has been reported that vitamin A content maintained relatively high levels in the gill of lampreys (*Lampetra japonica*) (10). Thus, it is reasonable for us to hypothesize that there might be a possible relationship between vitamin A and fish gill immunity, which has been investigated in this study.

The gill immunity is closely related to the various immunological parameters, namely, antibacterial peptides, lysozyme, and cytokines mediated by nuclear factor kappa B (NF-κB) and target of rapamycin (TOR) signaling pathways in fish (11). However, no studies so far have analyzed the influence of vitamin A in fish gill immune function and potential underlying mechanisms. In rat, it was shown that vitamin A can strengthen the phagocytic ability of neutrophils (12). To our knowledge, neutrophils could be activated by cytokines in animals (13). In addition, Berry et al. (14) also observed that vitamin A inhibited insulin responses in human hepatoma (HepG2) cells. In addition, inhibition of insulin substantially downregulated NF-κB transcriptional activities in the mouse skeletal muscle (15). In rat, Zorn and Sauro (16) reported that vitamin A promoted nuclear protein kinase C (nPKC) in splenic cells. It was found that nPKC could effectively contribute to mammalian target of rapamycin (mTOR) activation in feline cardiomyocytes (17). These findings suggested a potential relationship between vitamin A and gill immunity in fish gills, and further investigation might be needed.

Immune function is strongly dependent on the immune organ structural integrity (18). It is well known that fish gill structural integrity can be tightly linked with cellular integrities (oxidative damage vs. antioxidant ability and apoptosis) and the cell-to-cell tight junction (TJ) complexes, which generally can be modulated by signaling NF-E2-related factor 2 (Nrf2) (19), p38 mitogen-activated protein kinase (p38MAPK) (20), and myosin light chain kinase (MLCK) (21), respectively. However, whether vitamin A can significantly affect fish gill physical barrier function and mechanisms have not yet been studied. It has been demonstrated that vitamin A inhibited the inducible nitric oxide synthase (iNOS) expression in the rat microglia (22). Moreover, studies have shown that decreased iNOS expression could inhibit osteocyte apoptosis in human (23) and diminish the Nrf2 activation in THP-1 human monocytic cells (24). Additionally, in the human skin, Varani et al. (2000) demonstrated that vitamin A substantially attenuated the levels of matrix metalloproteinases (MMPs) (25). Interestingly, it was found that a decrease in MMP activity caused a marked increase in the expression of the rat brain TJ protein zonula occludens-1 (ZO-1) and occludin (26). Meanwhile, vitamin A could also promote androgen content in rats (27). Androgen can lead to a reduction in the levels of MLCK mRNAs in human prostate cancer cells (28). Overall, it has been revealed that a possible relation exists between vitamin A and gill physical barrier in fish, which is worth investigating.

The present study is a continuation of our previous study (9). Here, we have examined the potential influence of vitamin A on the gill mucosal immune components, cytokines, TJ proteins, antioxidant enzymes, and related signaling factors, Nrf2, p38MAPK, MLCK, NF-κB, and TOR in fish, which might provide an important theoretical basis for revealing the actions and mechanisms of vitamin A in significantly improving gill health of fish. In addition, optimal vitamin A levels based on the gill health-related parameters for on-growing grass carp were also evaluated.

## 2 Materials and Methods

### 2.1 Experimental Diet Preparation and Feeding Trial

Basal diet formulation (indicated in **Table S1**) was similar to that used in our previous study (9). Retinyl acetate (500,000 IU/g) was supplemented to six experimental diets at concentrations of 0 (unsupplemented control), 600, 1,200, 1,800, 2,800, and 3,800 IU/kg diet, and the corn starch content decreased accordingly. The final concentration of vitamin A in each treatment was found to be 18.69 (vitamin A deficiency, unsupplementation), 606.8, 1,209, 1,798, 2,805, and 3,796 IU/kg diet measured by high-performance liquid chromatographic assay. The finished diets were stored in -20°C refrigerator.

The animal study protocol was reviewed and approved by The Animal Care and Use Committee of Sichuan Agricultural University. Healthy grass carp were purchased from a local fishery (Sichuan) and were fed a vitamin A-insufficient diet for 2 weeks to eliminate the stored vitamin A in the body, and then 540 fish (262.02 ± 0.45 g) were randomly divided into 18 different cages. A 100-cm diameter tray was then placed at the bottom of each cage to collect the leftover feed. The fish were fed four times a day for 10 weeks. Experimental water temperature, pH, and dissolved oxygen were measured at 28°C ± 2°C, 7.0 ± 0.2, and not less than 6.0 mg/L, respectively. This study was conducted under natural light and dark cycle. At the end of the growth trial, the mean weight and SD from Groups 1 to 6 were 836.09 ± 24.54, 888.01 ± 23.41, 939.54 ± 13.34, 996.67 ± 32.18, 906.17 ± 3.69, and 848.89 ± 16.68 g, respectively, which clearly indicated that vitamin A with optimal dose could effectively promote the growth of fish (9).

### 2.2 Challenge Test and Sample Collection

By the end of the growth trial, 15 fish with similar weight were selected from each treatment group and transferred to the new cages. After acclimatization for the initial 5 days, fish were infected with *F. columnare* by immersion (from College of Veterinary Medicine, Sichuan Agricultural University, China) at a concentration of 1.0 * 10^8^ colony-forming units (CFU)/ml for 3 h and then were returned to each cage for feeding for 3 days. During this period, each treatment group was still fed the corresponding diet consistent with the 10-week growth trial. The *F. columnare* culture used was similar to the method by Shoemaker et al. (29). Briefly, *F. columnare* stock that was previously stored at -80°C was retrieved and grown in medium with shaking at 100 revolutions per minute (rpm) on an orbital shaker at 28°C for 24 h and thereafter expanded again. The concentration was about 10^11^ CFU/ml counted by the plate counting method and diluted to the corresponding concentration. The infection concentration was selected before (data not shown), which was only sufficient to activate the immune system but did not result in the death of the fish. The fish were monitored every day, and as expected, no fish died during the experiment. At the end of the infection trial, all fish were anesthetized according to Chen et al. (11). Fish were considered morbid when gill filaments were congested, swollen, and covered by profuse mucus (30, 31). According to a scoring system based on the method of Song et al. (2), gill rot morbidity was also evaluated. The different parts of the gills were preserved for histological examination. Thereafter, the rest of the portion of gills were quickly sampled, frozen, and stored at -80°C.

### 2.3 Analysis

#### 2.3.1 Histological and Biochemical Analysis

Histological examination was carried out by the method described in our previous study (32). Gill samples were homogenized in 10 volumes of cold physiological saline on ice and then centrifuged at 6,000 g for 20 min at 4°C. Thereafter, the supernatant was collected for further analysis. The activities of gill lysozyme and acid phosphatase (ACP) were analyzed by the method described by Chi et al. (33). The contents of reactive oxygen species (ROS), protein carbonyl (PC), complement 3 (C3), and C4 were analyzed according to the methods indicated by Chen et al. (11). The levels of anti-superoxide anion (ASA) and anti-hydroxyl radical (AHR) were tested based on the methods described by Hong et al. (34). Glutathione peroxidase (GPx), glutathione-*S*-transferase (GST), catalase (CAT), and glutathione reductase (GR) levels were measured according to the methods indicated by Peixoto et al. (35). Analyses of malondialdehyde (MDA), superoxide dismutase (SOD) activity, and reduced glutathione (GSH) contents were done based on the methods described by Zhang et al. (36).

#### 2.3.2 Quantitative Real-Time PCR

The total RNA isolation, reverse transcription, and quantitative real-time PCR were conducted based on the protocols described previously by our group (11). Gill total RNA was isolated by an RNAiso Plus (Takara, Dalian, China) and subjected to DNAse I treatment. Thereafter, RNA was reverse transcribed to cDNA by a PrimeScript™RT reagent Kit (Takara, Dalian, China). Quantitative real-time PCR was performed by specific primers designed according to the sequences of grass carp (**Table S2**). Then, based on the results of our preliminary experiment related to the evaluation of internal control genes (data not shown), β-actin gene was selected as an internal control. All the primer amplification efficiencies were approximately found to be 100%. The results were analyzed according to the 2^−ΔΔCT^ method by Livak and Schmittgen (37).

### 2.4 Statistical Analysis

The data were subjected to one-way analysis of variance (ANOVA) and the Duncan’s multiple-range test to analyze the significant differences among the treatment groups at *P* < 0.05 by SPSS 20.0 (SPSS Inc., Chicago, IL, USA). The optimal vitamin A levels for gill health indicators of fish were analyzed by using a quadratic regression model.

## 3 Results

### 3.1 Prevalence and Pathological Results of Gill Rot in Fish After *F. columnare* Infection

**Figure 1:**
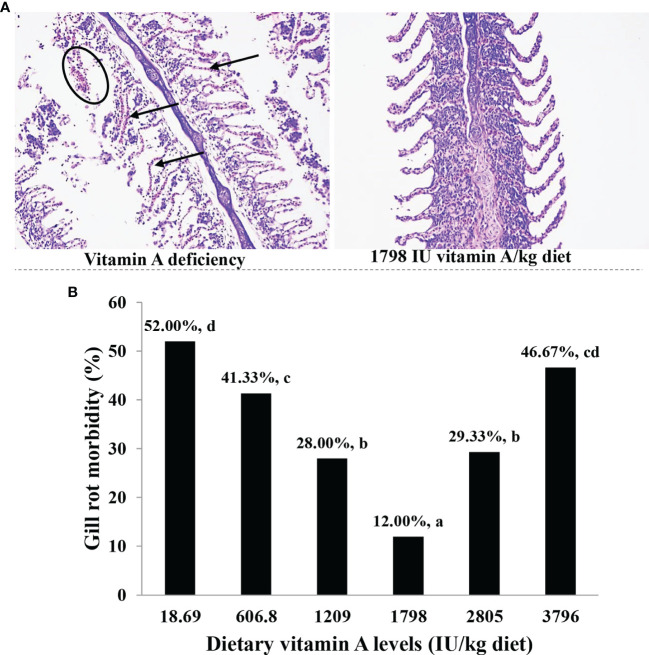
Gill histology **(A)** and gill rot morbidity **(B)** of on-growing grass carp (*Ctenopharyngodon idella*) fed diets containing the various vitamin A levels for 10 weeks and then infected by *F. columnare*. In panel **(A)**, the circle represents erosion, and the arrow indicates congestion, which represents six fish in each of the treatment groups. In panel **(B)**, the values represented are means of 15 fishes in each treatment, and different letters indicate significant differences (*P* < 0.05). Gill rot morbidity was estimated for every fish according to the standard rating system (higher scores indicate more serious effects) and finally calculated by weighted arithmetic mean.

As shown in **Figure 1**, when the dietary vitamin A level reached 1,798 IU/kg diet, the prevalence of gill rot was significantly found to be reduced to the minimum (12%) (*P* < 0.05). At the same time, the pathophysiological inspections indicated that vitamin A deficiency caused significant capillary hematoma and gill damage of fish infected by *F. columnare*.

### 3.2 Main Parameters of Gill Immune Function

#### 3.2.1 Antibacterial-Related Components in Fish Gill

**Figure 2:**
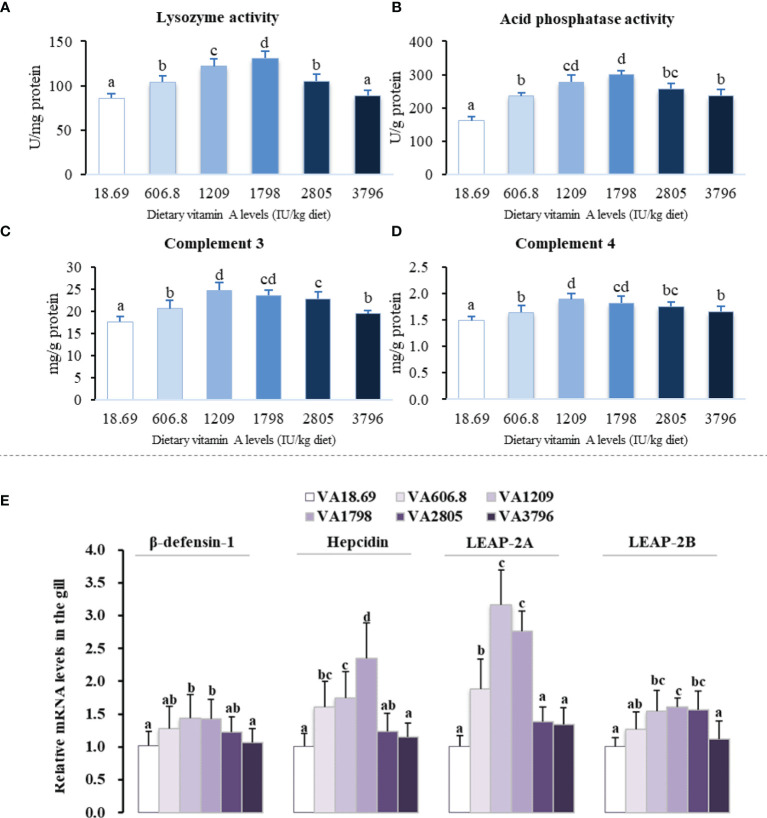
Antibacterial compound activity or contents **(A–D)** and relative mRNAs of antibacterial peptides **(E)** in the gill of on-growing grass carp fed diets containing various vitamin A levels for 10 weeks, which were then infected by *F. columnare*. The values shown are means (n = 6); error bars indicate SD. Different letters indicate the significant differences (*P* < 0.05). β-actin was used as an internal control gene. LEAP, liver-expressed antimicrobial peptide.

The various antibacterial-related components of gills are shown in **Figure 2**. The activity of lysozyme was the highest in fish fed the 1,798 IU vitamin A/kg diet but found to be the lowest in fish fed insufficient vitamin A and in the 3,796 IU vitamin A/kg diet group (*P* < 0.05). ACP activity was noted to be increased with an increase of the dietary vitamin A level to 1,798 IU/kg and then reduced with further increase of the vitamin A level (*P* < 0.05). In the gills, the levels of both C3 and C4 components were the lowest in fish fed the vitamin A-deficient diet, and these were increased in the 1,209 IU/kg diet but decreased thereafter upon exposure to the higher vitamin A levels (*P* < 0.05). The expression of β-defensin-1 and liver-expressed antimicrobial peptide 2B (LEAP-2B) mRNAs increased gradually and attained significant levels when vitamin A in the diets have reached 1,209 IU/kg and 1,798 IU/kg, respectively, but decreased thereafter. Moreover, it was observed that with an increase in dietary vitamin A level to 1,798 IU/kg, the levels of hepcidin mRNAs gradually increased and then decreased thereafter (*P* < 0.05). The expression of gill LEAP-2A mRNAs of fish fed the 1,209 and 1,798 IU vitamin A/kg diets was found to be relatively higher than that of other groups (*P* < 0.05), and there were no significant differences between these two groups (*P* > 0.05).

#### 3.2.2 The Expression of mRNAs of Gill Inflammatory Cytokines and Related Signaling Molecules

**Figure 3:**
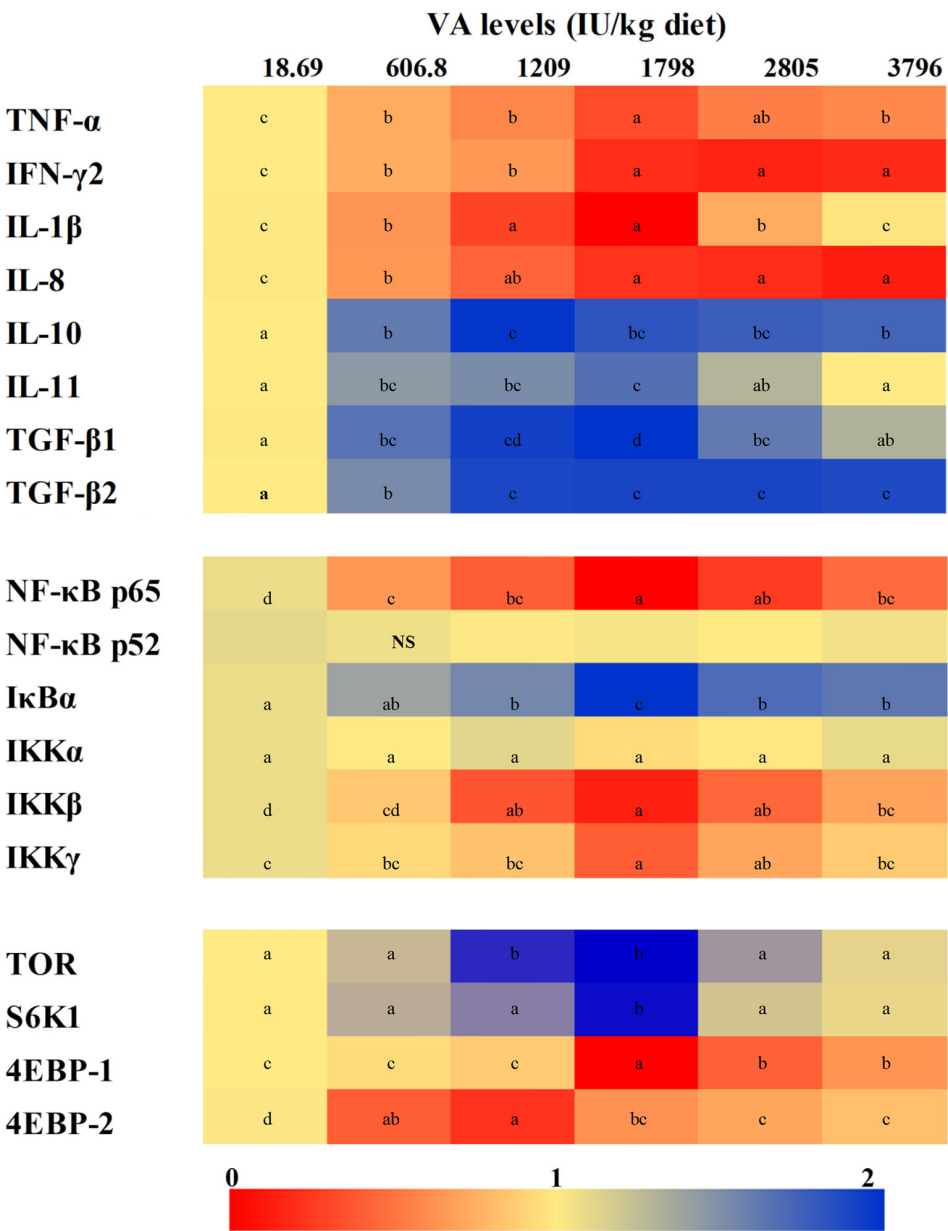
Heatmap for the expression of relative mRNAs of different cytokines and related signaling factors in the gill of on-growing grass carp fed diets containing various vitamin A levels for 10 weeks, which were then infected with *F. columnare*. The values shown are means (n = 6). The different letters indicate the significant differences (*P* < 0.05). β-actin was used as an internal control gene. TNF-α, tumor necrosis factor α; IFN-γ2, interferon γ2; IL, interleukin; TGF, transforming growth factor; NF-κB, nuclear factor κB; IκB, inhibitor κB; IKK, IκB kinase; TOR, target of rapamycin; S6K1, S6 kinase 1; 4EBP, eukaryotic translation initiation factor 4E-binding protein. NS, not significant difference.

The expression of gill inflammatory cytokines and related signal factor mRNAs has been shown in **Figure 3**. The expression of *tumor necrosis factor α (TNF-α)* mRNAs decreased significantly with the level of vitamin A to 1,798 IU/kg diet (*P* < 0.05) and then slowly increased. The expression of mRNAs of *interferon γ2 (IFN-γ2)* and *interleukin-8 (IL-8)* decreased when vitamin A levels were enhanced to 1,798 and 1,209 IU/kg diet (*P* < 0.05), respectively. The expression of *IL-1β* mRNAs was found to be the highest in fish fed the vitamin A-deficient and 3,796 IU/kg diets and was the lowest in fish fed the 1,209–1,798 IU vitamin A/kg diet (*P* < 0.05). The expression of *IL-10* mRNAs increased significantly with dietary vitamin A level to 1,209 IU/kg (*P* < 0.05) and then slowly decreased with an increase of vitamin A level. The expression of both *IL-11* and *transforming growth factor β1 (TGF-β1)* mRNAs rose gradually when the dietary vitamin A level reached 1,798 IU/kg but decreased thereafter (*P* < 0.05). The expression of *TGF-β2* mRNAs increased significantly with increasing levels of dietary vitamin A to 1,209 IU/kg (*P* < 0.05) but stabilized thereafter (*P* > 0.05). At the same time, the expression of *NF-κBp65* mRNAs decreased significantly in 1,798 IU vitamin A/kg diet (*P* < 0.05) and then slowly increased with further increase in the levels of vitamin A. However, dietary vitamin A exhibited no significant effect on the mRNA level of *NF-κB* p52 (*P* > 0.05). The expression of gill *IκBα* mRNAs increased significantly with the increase in the level of vitamin A to 1,798 IU/kg diet and then decreased substantially (*P* < 0.05). The expression of *IκB kinase β (IKKβ)* and *IKKγ* mRNAs decreased significantly when the dietary vitamin A level had reached 1,798 IU/kg (*P* < 0.05) but gradually increased thereafter. However, the expression of gill *IKKα* mRNA was not significantly affected by the dietary vitamin A levels, but that of gill *TOR* mRNA level of fish fed the vitamin A 1,209 and 1,798 IU/kg concentrates was found to be higher than those of other groups (*P* < 0.05). In addition, fish fed the dietary vitamin A 1,798 IU/kg showed the higher expression of *ribosome protein S6 kinase 1 (S6K1)* mRNA level than other groups (*P* < 0.05). The expression of *eIF4E-binding protein 1 (4E-BP1)* mRNAs was significantly reduced with the increase in the dietary vitamin A level up to 1,798 IU/kg and then increased proportionately with higher vitamin A levels (*P* < 0.05). The expression of *4E-BP2* mRNAs was found to be the highest for fish fed the vitamin A-deficient diet (*P* < 0.05) and were downregulated markedly with increasing dietary vitamin A levels to 1,209 IU/kg but then were significantly upregulated (*P* < 0.05).

### 3.3 Effect on the Main Parameters of Oxidation and Antioxidant Capacity

**Figure 4:**
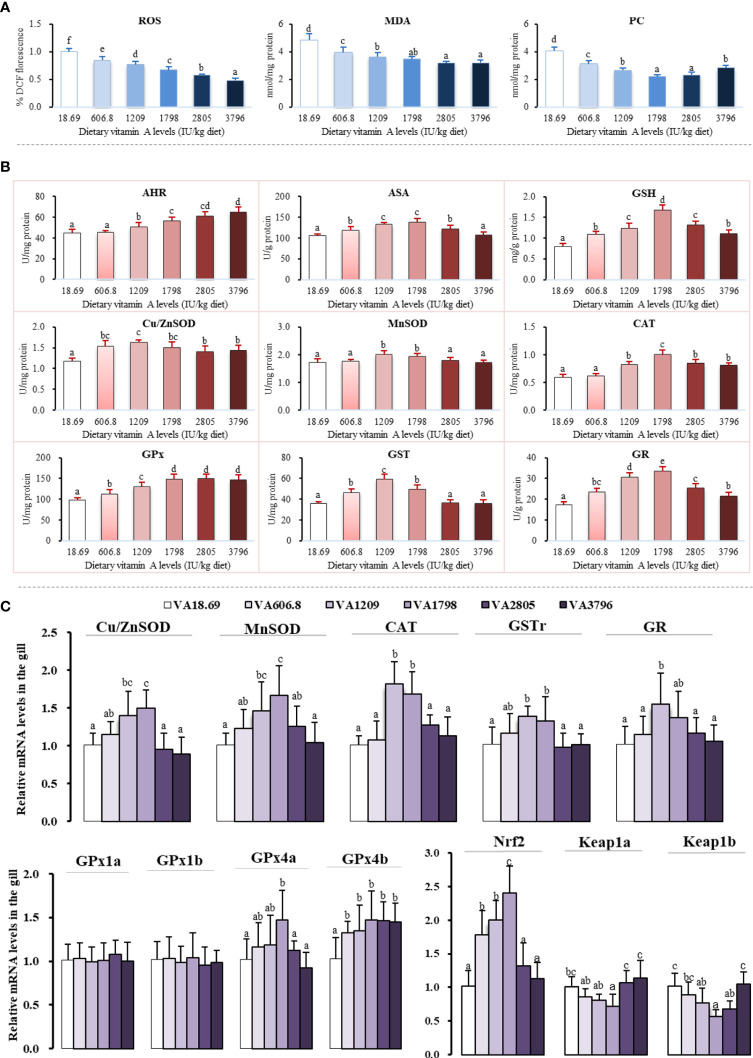
Potential effects of dietary vitamin A on oxidative damage **(A)**, antioxidant enzyme activities **(B)**, and relative expression of mRNAs of antioxidants and related signaling factors **(C)** in the gill of fish. The values shown are the means (n = 6); error bars indicate SD. The different letters indicate the significant difference (*P* < 0.05). β-actin was used as an internal control gene. ROS, reactive oxygen species; MDA, malondialdehyde; PC, protein carbonyl; AHR, anti-hydroxy radical; ASA, anti-superoxide anion; Cu/ZnSOD, copper/zinc superoxide dismutase; MnSOD, manganese superoxide dismutase; CAT, catalase; GPx, glutathione peroxidase; GST, glutathione-S-transferase; GR, glutathione reductase; GSH, glutathione.

The main parameters related to oxidation and antioxidation in gills have been shown in **Figure 4**. It was noted that ROS concentration was significantly decreased with 3,796 IU vitamin A/kg diet (*P* < 0.05). At the same time, gill MDA and PC contents of fish fed the vitamin A-lacking diet were found to be substantially higher than those of another supplementary vitamin A group (*P* < 0.05). With the dietary vitamin A level reaching 3,796 IU/kg diet, gill AHR capacity increased significantly (*P* < 0.05). The ASA and MnSOD activities of fish fed vitamin A 1,209–1,798 IU/kg concentrations were found to be higher than those of any group (*P* < 0.05). The activity of gill Cu/ZnSOD was noted to be gradually increased with the dietary vitamin A level to 1,209 IU/kg diet and then decreased with the further increase of the vitamin A level. The fish fed the 1,798 IU/kg vitamin A diet exhibited higher CAT and GR activities and GSH contents in their gills (*P* < 0.05). The gill GPx activity was noted to be significantly increased with the dietary vitamin A level to 1,798 IU/kg (*P* < 0.05), and with a further increase in the dietary vitamin A level, no significant differences were found (*P* > 0.05). However, with the dietary vitamin A level reaching 1,209 IU/kg, the activity of gill GST first increased significantly and then decreased significantly later (*P* < 0.05).

As shown in **Figure 4**, the expression of *Cu/ZnSOD, MnSOD, GPx4a,* and *GSTr* mRNAs slowly increased when the dietary vitamin A level reached 1,798 IU/kg and then decreased significantly with an increase of the vitamin A level (*P* < 0.05). The expression of *CAT* mRNA level of fish fed with the vitamin A 1209–1798 IU/kg diet was significantly higher than that of any other group (*P* < 0.05). In addition, compared with other vitamin A-supplemented diets, the expression of gill *GPx4b* mRNAs of fish fed lacking vitamin A diets was significantly reduced (*P* < 0.05), and there were no significant differences observed between these groups (*P* > 0.05). The expression of gill *GR* mRNAs increased significantly with an increase in the dietary vitamin A level to 1,209 IU/kg (*P* < 0.05) but gradually decreased since then. Dietary vitamin A exhibited no significant influence on the mRNA levels of *GPx1a* and *GPx1b* (*P* > 0.05).

Compared with other groups, fish fed the vitamin A 1798 IU/kg diet exhibited the highest level of *Nrf2* mRNA (*P* < 0.05). As the dietary vitamin A level reached 1,798 IU/kg, the levels of *Kelch-like ECH-related protein (keap1a)* mRNAs slowly declined and then was observed to increase significantly (*P* < 0.05). The expression of *keap1b* mRNAs slowly decreased with the vitamin A levels increasing to 1,798 IU/kg diet and then slowly increased with an increase of the vitamin A level.

### 3.4 Apoptosis-Related Gene Expression in the Fish Gill

**Figure 5:**
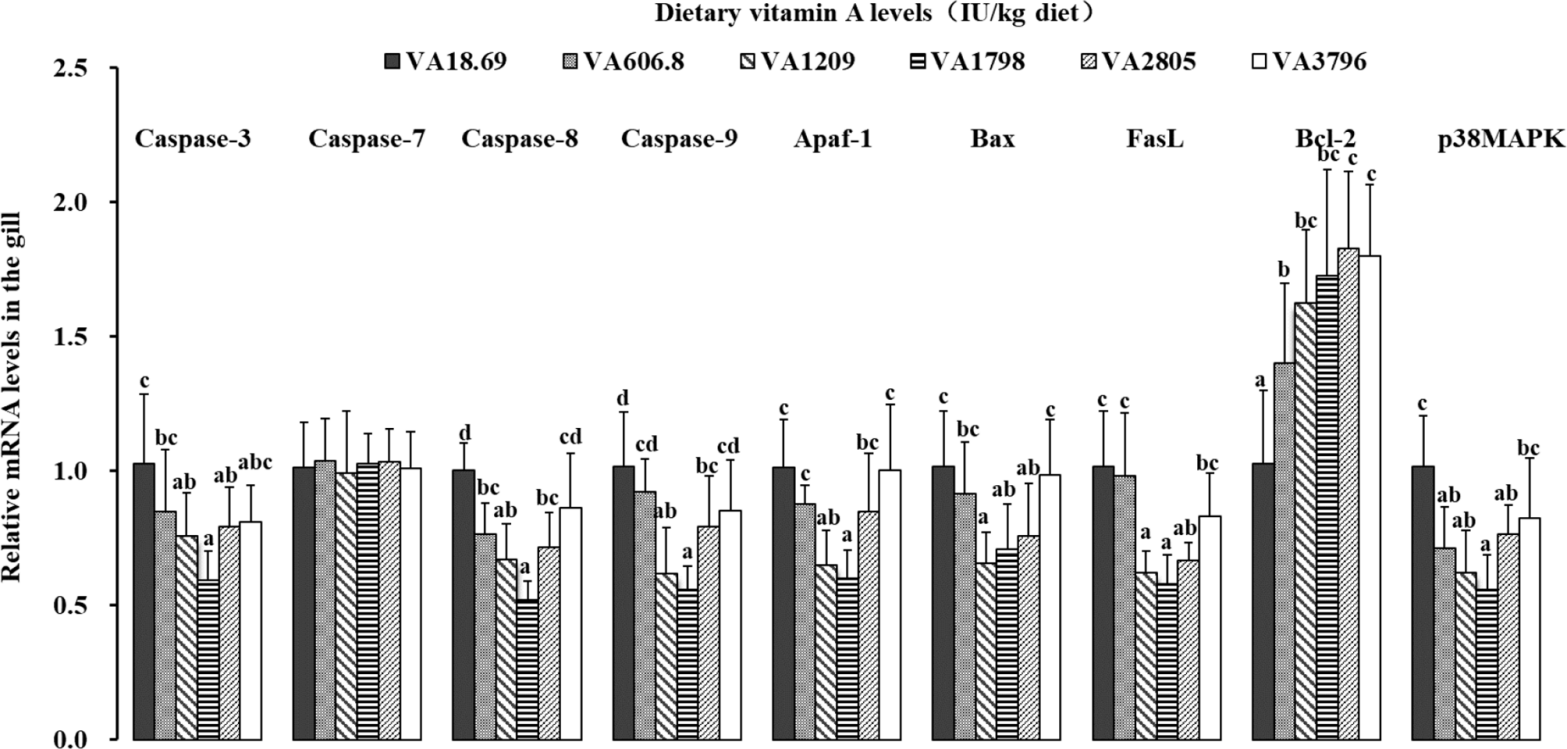
Relative expression of mRNAs of apoptosis-related factors in the gill of fish fed diets containing various vitamin A levels for 10 weeks, which were then infected by *F. columnare*. The values shown are the means (n = 6); error bars indicate SD. The different letters indicate the significant difference (*P* < 0.05). β-actin was used as an internal control gene. Apaf-1, apoptotic protease-activating factor 1; Bax, B-cell lymphoma protein 2-associated X protein; FasL, Fas ligand; Bcl-2, B-cell leukemia/lymphoma-2; p38MAPK, p38 mitogen-activated protein kinase.

As shown in the **Figure 5**, the levels of caspase-3 and Fas ligand (FasL) mRNAs slowly decreased when the dietary vitamin A level reached 1,798 IU/kg diet and then increased gradually. The mRNA levels of *caspase-8, caspase-9*, and *apoptotic protease-activating factor 1 (Apaf-1)* slowly decreased when the dietary vitamin A level reached 1,798 IU/kg diet and then significantly increased with further increase in vitamin A levels (*P* < 0.05). The expression of *B-cell lymphoma protein 2-associated X protein (Bax)* mRNAs decreased significantly when the dietary vitamin A level reached 1,209 IU/kg diet (*P* < 0.05) and thereafter slowly increased thereafter. The expression of *p38MAPK* mRNAs gradually decreased when the dietary vitamin A level reached 1,798 IU/kg diet and then slowly increased thereafter. The expression of *B-cell leukemia/lymphoma-2 (Bcl-2)* mRNAs slowly increased with vitamin A level to 2,805 IU/kg diet and thereafter reached a plateau thereafter. However, dietary vitamin A was not able to significantly alter the expression of *caspase-7* mRNAs in the fish gill.

### 3.5 Tight Junction Complexes and Myosin Light Chain Kinase mRNA Levels in the Fish Gill

**Figure 6:**
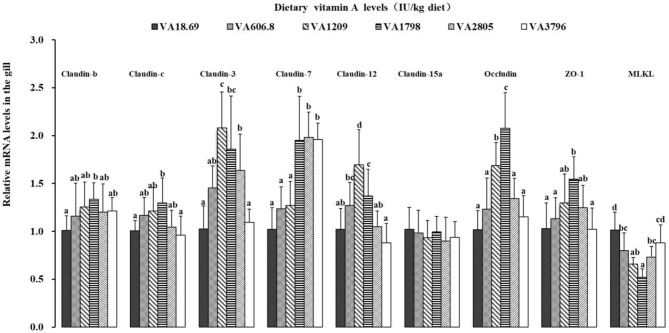
Relative expression of mRNAs of the tight junction-related factors in the gill of on-growing grass carp fed diets containing various vitamin A levels for 10 weeks, which were then infected by *F. columnare*. The values shown are the means (n = 6); error bars indicate SD. β-actin was used as an internal control gene. Different letters indicate the significant difference (*P* < 0.05). ZO-1, zonula occludens-1; MLCK, myosin light chain kinase.

The possible effects of vitamin A on the expression of related TJ complexes and *MLCK* mRNAs have been shown in **Figure 6**. The expression of *claudin-b, claudin-c*, and *ZO-1* mRNAs was increased slowly when the dietary vitamin A level reached 1,798 IU/kg and gradually decreased thereafter. The expression levels of *claudin-3* mRNAs were slowly increased with the increase in the dietary vitamin A level to 1,209 IU/kg (*P* < 0.05) and subsequently decreased thereafter. Fish fed dietary vitamin A 1,798, 2,805, and 3,796 IU/kg diets displayed relatively higher levels of *claudin-7* mRNA than those in other groups (*P* < 0.05), and no significant differences were found among these groups (*P* > 0.05). The expression of *claudin-12* and *occludin* mRNAs was increased significantly when the dietary vitamin A level reached 1,798 IU/kg and then decreased thereafter (*P* < 0.05). The expression of *MLCK* mRNAs was gradually decreased with dietary vitamin A level to 1,798 IU/kg and then significantly increased with higher vitamin A levels (*P* < 0.05). However, the expression of gill *claudin-15a* mRNAs was not significantly influenced by dietary vitamin A levels (*P* > 0.05).

## 4 Discussion

The current study was conducted using the same growth trial as described in our previous report (9), which was a portion of systematic research for analyzing the effects of vitamin A on the growth, immune, and barrier function in fish. Our previous findings have revealed that vitamin A promoted significant growth performance of grass carp (9). It is well known that, in fish, the growth performance is tightly related to gill health (18). Therefore, here, we have focused on examining the potential impact of vitamin A on gill health in fish.

### 4.1 Vitamin A Decreased Gill Rot Morbidity

In the current study, the rate of gill rot morbidity in the vitamin A-deficient diet (gill rot morbidity = 52%) was found to be significantly higher than that in optimum vitamin A administration (12%) when infected by *F. columnare*. The results indicated that vitamin A deficiency could significantly increase the morbidity of gill rot in fish infected by *F. columnare*, and optimal levels of vitamin A could effectively reverse this negative influence. In general, the health status of gill is tightly related to the immune and physical barrier in fish (18). Thus, we examined for the first time the possible alterations in immune and physical barrier in the gills of fish after vitamin A administration.

### 4.2 Vitamin A Enhanced the Immune Responses in Fish Gill

#### 4.2.1 Vitamin A Increased the Antibacterial Ability in Fish Gill

The antimicrobial peptides (like *hepcidin, β-defensin*, and *LEAP-2*), ACP, lysozyme, and complement (such as C3 and C4) have been found to be the critical antibacterial compounds in fish (11). Our results illustrated that dietary vitamin A deficiency decreased fish gill mRNAs of *hepcidin, β-defensin, LEAP-2A*, and *LEAP-2B*, activities of ACP and lysozyme, and contents of C3 and C4, whereas appropriate vitamin A could facilitate recovery of these observed decreases. In addition, no prior study until now has determined the influence of vitamin A on the antibacterial ability in fish gill. The negative influence of vitamin A deficiency on gill antibacterial ability might be partially mediated as a result of the damage of immune cell function. In chicken, it was reported that vitamin A deficiency can effectively impair peritoneal macrophage activity (38). To our knowledge, the macrophage is an important regulator of the antibacterial components in fish (39). As mentioned above, fish antibacterial ability has been linked to inflammation that can be regulated by the different cytokines. Accordingly, in this study, effects of vitamin A on gill inflammation of fish were investigated next.

#### 4.2.2 Vitamin A Mitigated Inflammation by Modulating Nuclear Factor-κB and Target of Rapamycin Signaling Cascades in Fish Gill

A precious study has shown that the inflammatory process can be mitigated by a decrease of pro-inflammatory cytokines, such as *IL-1β* and *TNF-α*, and an increase of anti-inflammatory cytokines, such as IL-10 and *TGF-β* (40). In the current study, dietary vitamin A significantly decreased the expression of pro-inflammatory cytokine mRNAs (*TNF-α, IFN-γ2, IL-1β*, and *IL-8*) but increased the levels of anti-inflammatory cytokine mRNAs (*IL-10, IL-11, TGF-β1*, and *TGF-β2*), thereby suggesting the mitigation of gill inflammatory response by vitamin A administration. The mechanisms through which vitamin A can influence the cytokine expression in fish are still unknown. To our knowledge, NF-κB and TOR are the critical signaling pathways involved in the regulation of the production of cytokines in fish. Thus, the potential influence of vitamin A on NF-κB and TOR signaling in fish gill was examined in this study.

NF-κB, including p65 and p52, plays a critical role in the expression of multiple genes that are associated with inflammation (41). It has been reported that the levels of both *IFN-γ2* and *TNF-α* mRNAs can be stimulated by NF-κB p65 in gills of fish (11). We also observed that in comparison with the dietary vitamin A deficiency, optimum vitamin A levels can reduce the expression of *NF-κB p65* mRNAs in fish gill, but *NF-κB p52* mRNAs were not affected by vitamin A levels. Correlation analysis revealed that the expression of both gill pro-inflammatory cytokine (*TNF-α, IFN-γ2*, and *IL-8*) and anti-inflammatory cytokine (*IL-10* and *TGF-β2*) mRNAs showed positive and negative correlation with *NF-κB p65*, respectively, thus implying that the effects of vitamin A might be partly through inhibiting the levels of NF-κB p65 (not *NF-κB p52*) mRNAs to inhibit the levels of pro-inflammatory and promote that of anti-inflammatory cytokines as mentioned above, thereby leading to the mitigated fish gill excessive inflammatory response. Additionally, in fish, IκBα can regulate NF-κB levels by inhibiting the translocation of NF-κB from the cytoplasm to the nucleus (42). A previous study using *in vitro* mouse embryonic fibroblasts (*MEFs*) indicated that the increase of *IκBα* inhibited NF-κB p65 nuclear translocation (43). In the current study, dietary vitamin A was found to markedly promote the expression of gill *IκBα* mRNAs. Furthermore, correlation analysis revealed a significant negative correlation between *NF-κB p65* and *IκBα* (**Table S3**), thereby suggesting that vitamin A-induced downregulation of the levels of *NF-κB p65* mRNAs might be in part associated with an increase of *IκBα* mRNAs. Moreover, Li et al. (44) have reported that *IκBα* could be phosphorylated and degraded by IKK, which consists of three distinct subunits (α, β, and γ), thus causing NF-κB activation in MEFs. In the current study, fish fed the optimum vitamin A diet downregulated the expression of both *IKKβ* and *γ* mRNAs in the fish gill. Correlation analysis indicated that the levels of mRNAs of *IκBα* were negatively correlated with the mRNAs of *IKKβ* and *IKKγ* (**Table S3**), thus demonstrating that vitamin A-upregulated *IκBα* mRNA level may be in part associated with the reduced levels of *IKKβ* and *IKKγ* mRNAs.

Interestingly, dietary vitamin A levels did not alter the levels of *IKKα* mRNAs, which may be related to a disparate impact of vitamin A on different NF-κB signaling pathways. It is well known that NF-κB signaling can be divided into two, namely, canonical and non-canonical signaling pathways. Canonical signaling primarily relies on activating IKK (β and γ), and degradation of IκBα can then cause NF-κBs (p65/p50/c-Rel) to translocate into the nucleus. Inversely, non-canonical NF-κB activation is dependent on activating IKKα to cause the formation of p52/RelB dimers in cancer cells (45). Here, we observed that the expression of both *IKKα* and *NF-κB p52* mRNAs was not substantially affected by vitamin A levels, which indicated that vitamin A-modulated cytokines mentioned above may be predominantly related to NF-κB canonical signaling, not non-canonical signaling, pathway. However, the underlying mechanisms need further investigations.

In addition, a central role for TOR and its downstream targets S6K1 and 4E-BPs in regulating inflammatory responses has been established (46). In this study, it was found that the dietary vitamin A increased the levels of *TOR* and *S6K1* mRNAs, whereas it decreased the expression of *4EBP1* and *4EBP2* mRNAs in fish gill. Correlation analysis indicated that the levels of both the pro-inflammatory (*IL-1β*) and anti-inflammatory cytokine (*IL-11* and *TGF-β1*) mRNAs displayed a negative and positive correlation with *TOR* and *S6K1*, respectively (**Table S3**), thus indicating that *TOR/S6K1* signaling might also contribute to the vitamin A-mediated regulation of the gill inflammatory response of the fish.

Taken together, in fish gill, vitamin A could effectively enhance the antibacterial ability that might be caused by inhibition of IKK(β,γ)/IκBα/NF-κB p65-mediated NF-κB canonical signaling pathway (not IKKα/NF-κB p52-mediated non-canonical signaling) and TOR/S6K1 signaling to mitigate an inflammatory response, which finally results in the attenuation of immune function. In addition to an optimal immune function, fish gill health also largely depends on its physical barrier function. Therefore, it will be important for us to investigate the impact of vitamin A on the physical barrier function in gills.

### 4.3 Vitamin A Maintained Gill Physical Barrier Function of Fish Infected by *F. columnare*

#### 4.3.1 Vitamin A Increased the Antioxidant Capacity in Fish Gills

It is well known that gill structural integrity is a critical element for maintaining fish gill physical barrier. A previous study has shown that the gill structural integrity could be easily affected by oxidative damage caused by ROS, especially lipid and protein oxidation damage (47). Generally, MDA and PC are two well-known lipid peroxidation and protein oxidation indicators, respectively (48). In our current study, in comparison to vitamin A deficiency, optimal vitamin A levels were found to significantly suppress the ROS, MDA, and PC concentrations in the fish gill, thus suggesting that vitamin A decreased the oxidative damage of fish gill. Moreover, superoxide radical (O_2_^•−^) and hydroxyl radical (•OH-) are two of the main toxic ROS moieties that are involved in oxidative damage in fish (49). A prior study has indicated that ASA and AHR activities can serve as two important indicators to determine the O_2_^•−^ and •OH-scavenging abilities, respectively (50). In the current study, optimal levels of vitamin A significantly increased AHR and ASA capacity, thereby indicating that vitamin A-decreased ROS production might be partly associated with a concomitant increase of the free radical-scavenging capacity in fish gill. Besides, fish own antioxidant system, namely, non-enzymatic (such as GSH) and enzymatic (like SOD, CAT, GPx, GST, and GR) antioxidants can be employed to protect the gill resistance from oxidative stress (51). Here, we observed that in fish gill, optimum vitamin A increased GSH content and activities of T-SOD, Cu/ZnSOD, GPx, CAT, GST, and GR, thereby indicating that vitamin A increased fish gill antioxidant capacity. Commonly, antioxidant enzyme activities are in part dependent on their gene expression level, which can be primarily regulated by Nrf2 signaling (52). Accordingly, we next proceeded to check the impact of vitamin A on the antioxidant enzyme mRNAs and Nrf2 signaling.

Here, we demonstrated that dietary optimum vitamin A resulted in a substantial increase in the expression of *Cu/ZnSOD, MnSOD, CAT, GPx4a, GPx4b, GSTr*, and *GR* mRNAs. Furthermore, the results showed that antioxidant activities (MnSOD, GPx, GST, and GR) were positively correlated with levels of their corresponding mRNAs (MnSOD, GPx4b, GSTr, and GR) in fish gill (**Table S3**), thus implying that the increase in aforesaid antioxidant enzyme activities can be at least partly attributed to the fact that vitamin A effectively upregulated corresponding gene expressions in the fish gill. Furthermore, optimum vitamin A increased the expression of *Nrf2* mRNAs. Positive correlation was also found between mRNA levels of antioxidant enzymes (*CuZnSOD, MnSOD, GPx4a, GPx4b, GSTr*, and *GR*) and *Nrf2* (**Table S3**), thereby implying that increased antioxidant enzyme mRNAs by vitamin A might be partly related to the upregulation of Nrf2 in fish gill. In addition, it has been well established that under normal conditions, Nrf2 is bound by Keap1 in the cytoplasm and can translocate to the nucleus when exposed to stress. In mice, Keap1 knockout could lead to constitutive Nrf2 activity in embryonic fibroblasts, which can result in an increased mRNA level of *GSTp1* (53). Interestingly, the results from the current study also indicated that optimal vitamin A markedly decreased the levels of both *Keap1a* and *Keap1b* mRNAs. A negative correlation was found between *Nrf2* and *Keap1a* mRNA levels in fish gills (**Table S3**), thus implying that the increase of *Nrf2* mRNAs may be in part related to the suppression in the levels of *Keap1a* mRNAs in fish gills by vitamin A. As a result, cell apoptosis might occur when the cells are subjected to oxidative damage. Thus, we further investigated the potential influence of vitamin A on apoptosis signaling in fish gills.

#### 4.3.2 Vitamin A Inhibited Cellular Apoptosis in Fish Gills

Usually, apoptosis functions as a normal physiological process to maintain cellular homeostasis. However, a previous study has demonstrated that excessive apoptosis can result in a severe loss of gill structural integrity (54). It is well known that caspases play a vital role in the process of apoptosis, and the downstream apoptosis executioner caspases like caspases 3 and 7 represent the degradation phase of apoptosis in fish (55). In this study, optimal dietary vitamin A levels were found to significantly downregulate the expression of gill *caspase-3* mRNAs, whereas the expression of *caspase-7* mRNAs was not affected by vitamin A treatment. The result suggested that in fish gills, vitamin A deficiency could induce cellular apoptosis through affecting caspase-3 (but not *caspase-7*). This result might be partly related to the adhesion action of vitamin A. A prior study demonstrated that, in rat, vitamin A can promote cell adhesion-related protein expression and thus enhance the cell–cell adhesion (56). Moreover, it has been reported that *caspase-3* can inhibit cell adhesion at a faster rate compared to caspase-7 in mice (57). In addition, the regulation of *caspase-3* mRNA level may be related to the upstream initiator *caspase-8* and *caspase-9*. For instance, a study in fish has revealed that an increase in the levels of *caspase-8* and *caspase-9* mRNAs could promote apoptosis (58). Our results demonstrated that appropriate vitamin A decreased the expression of both *caspase-8* and *caspase-9* mRNAs in fish gills. The correlation analysis showed that *caspase-3* mRNAs were positively correlated with the levels of *caspase-8* and *caspase-9* mRNAs (**Table S3**), which indicated that vitamin A-induced downregulation of the expression of *caspase-3* mRNA might be partly attributed to a reduction in both *caspase-8*-regulated extrinsic and *caspase-9*-regulated intrinsic pathways in the fish gill. Moreover, caspase activation could be regulated by the various cellular factors. For example, a previous study has reported that the induction of the caspase-8-mediated extrinsic pathway could be initiated by the binding of specific cytokine ligands [such as Fas ligand (FasL)] (59). Moreover, activation of the Bcl-2 family member Bax can cause a rapid release of cytochrome c and can then bind Apaf-1, thus forming the apoptosome and activating caspase-9 (60). It was reported that downregulation of pro-apoptotic molecules Bax and FasL and upregulation of anti-apoptotic molecule Bcl-2 could effectively inhibit caspase-dependent apoptosis in human osteosarcoma cells (60). Our results indicated that optimum vitamin A diet resulted in increased expression of anti-apoptotic protein *Bcl-2* and the remarkable decline in the levels of pro-apoptotic members *FasL, Bax*, and *Apaf-1* mRNAs in the fish gill. Correlation analysis showed that the levels of *caspase-8* and *caspase-9* mRNAs were positively correlated with FasL mRNAs and *Bax* and *Apaf-1* mRNAs, respectively (**Table S3**). This finding implied that vitamin A-induced decrease in the expression of *caspase-8* mRNAs might be partly due to the downregulation of pro-apoptotic protein *FasL* mRNA level, while the reduction of *caspase-9* mRNA level might be partly attributed to decreasing the expression of pro-apoptotic member mRNAs like *Bax* and *Apaf-1*. Besides, a critical role of p38MAPK signaling in apoptosis has emerged. A previous study has demonstrated that the decrease of p38MAPK activation decreased cardiomyocyte apoptosis in rabbits (61). The data from our study indicated that optimal vitamin A downregulated the levels of *p38MAPK* mRNAs. Further analysis revealed that the levels of both *caspase-8* and *caspase-9* mRNAs were positively correlated with p38MAPK mRNAs (**Table S3**), thus implying that vitamin A downregulated the expression of both *caspase-8* and *caspase-9* mRNAs, which might be in part linked to the decrease of *p38MAPK* mRNAs. Taken together, our findings indicated that vitamin A could effectively suppress fish gill apoptosis by inhibition of caspase-8-dependent extrinsic pathway that in part associated with the decrease of Apaf1 mRNAs and caspase-9-mediated intrinsic pathway that can be partially attributed to the downregulated mRNA levels of the various pro-apoptotic members (Bax and Apaf-1), and signaling molecule p38MAPK also contributed to the regulation of apoptosis signaling in the fish gill. In EpH4 cell lines, apoptosis has been reported to be also involved in the disruption of TJs (62). Thus, we further investigated the potential actions of vitamin A on the TJ complex in fish gills.

#### 4.3.3 Vitamin A Enhanced the Fish Gill Tight Junction Complex Integrity and the Effect Was Partially Associated With the Decrease of Myosin Light Chain Kinase Signaling

TJs like claudins, occludin, and ZO-1 can play important roles in maintaining the polarity of epithelial cells (63). The current study demonstrated that optimal vitamin A upregulated the expression of *claudins (-3, -7, -b, -c, -12), occludin*, and *ZO-1* mRNAs, thus suggesting that vitamin A enhanced the gill barrier function of the fish. Interestingly, *claudin-15a* mRNA level was not altered by vitamin A. This result may be in part associated with the action of vitamin A on the phospholipid. A previous study has revealed that vitamin A could promote the surfactant phospholipid synthesis in rat (64). In our previous study, phospholipid was found to exhibit no influence on the mRNAs of *claudin-15* in fish (65). Furthermore, the action of vitamin A on the TJ complex might be partially related to its impact on MLCK. In Caco-2_BBe_ cells, an increase of *MLCK* levels caused a loss of ZO-1 (66). Here, we observed that optimal vitamin A decreased the expression of MLCK mRNAs in fish gill. Further correlation analysis indicated that fish gill *claudin (-b, -c*, and *-3*), *occludin*, and *ZO-1* mRNAs were negatively related to the levels of *MLCK* mRNAs (**Table S3**), thus suggesting that the increase of TJ mRNAs by vitamin A may be partly mediated due to the decrease in the expression of *MLCK* mRNAs.

### 4.4 Vitamin A Requirements of On-Growing Grass Carp

**Figure 7:**
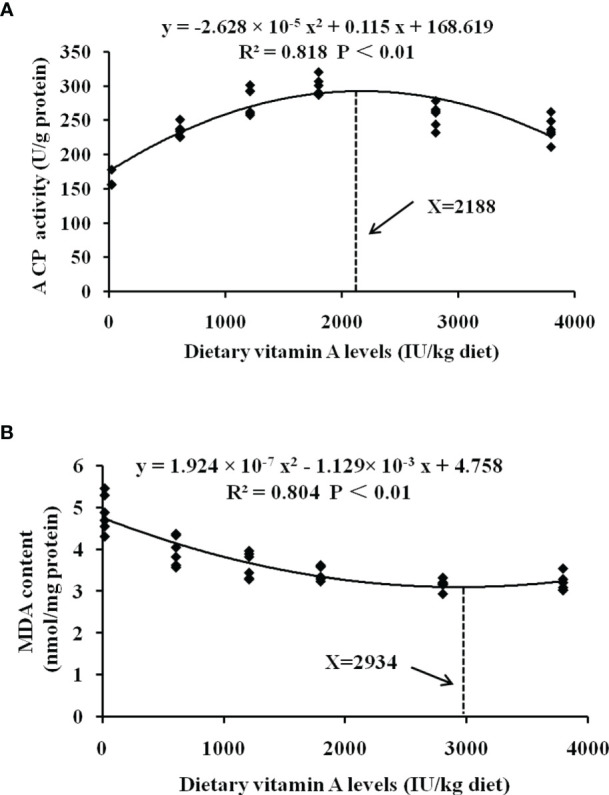
Quadratic regression analysis of MDA content **(A)** and ACP activities **(B)** for grass carp fed diets containing various vitamin A levels for 10 weeks. MDA, malondialdehyde; ACP, acid phosphatase.

Intensive aquaculture poses a significant risk to fish gill, and thereby maintaining gill health is very important. Thus, it is necessary to evaluate the vitamin A requirements for maintaining fish health. A reduction in morbidity is often used to assess the overall beneficial effects of the nutrients, while key immunological factors like ACP and structural integrity key markers like MDA are commonly used to evaluate the potential effects of the different nutrients on immunity and organ structural integrity, respectively. Thus, we evaluated the nutritional requirements of vitamin A in grass carp based on the gill rot morbidity, ACP, and MDA. To protect the fish against gill rot morbidity, ACP activity, and MDA content in the gill, the optimal vitamin A levels for grass carp (262–997 g) were found to be 1,991 (Y = 8.789 × 10^-6^ x^2^ -0.035x + 55.147, R² = 0.893, *P* < 0.05), 2,188, and 2,934 IU/kg diet, respectively (**Figure 7**). All the vitamin A requirements based on the gill healthy indices were observed to be significantly higher than the growth requirement (1,929 IU/kg) (9), thus suggesting that more vitamin A might be required for maintaining fish gill health.

## 5 Conclusions

**Figure 8:**
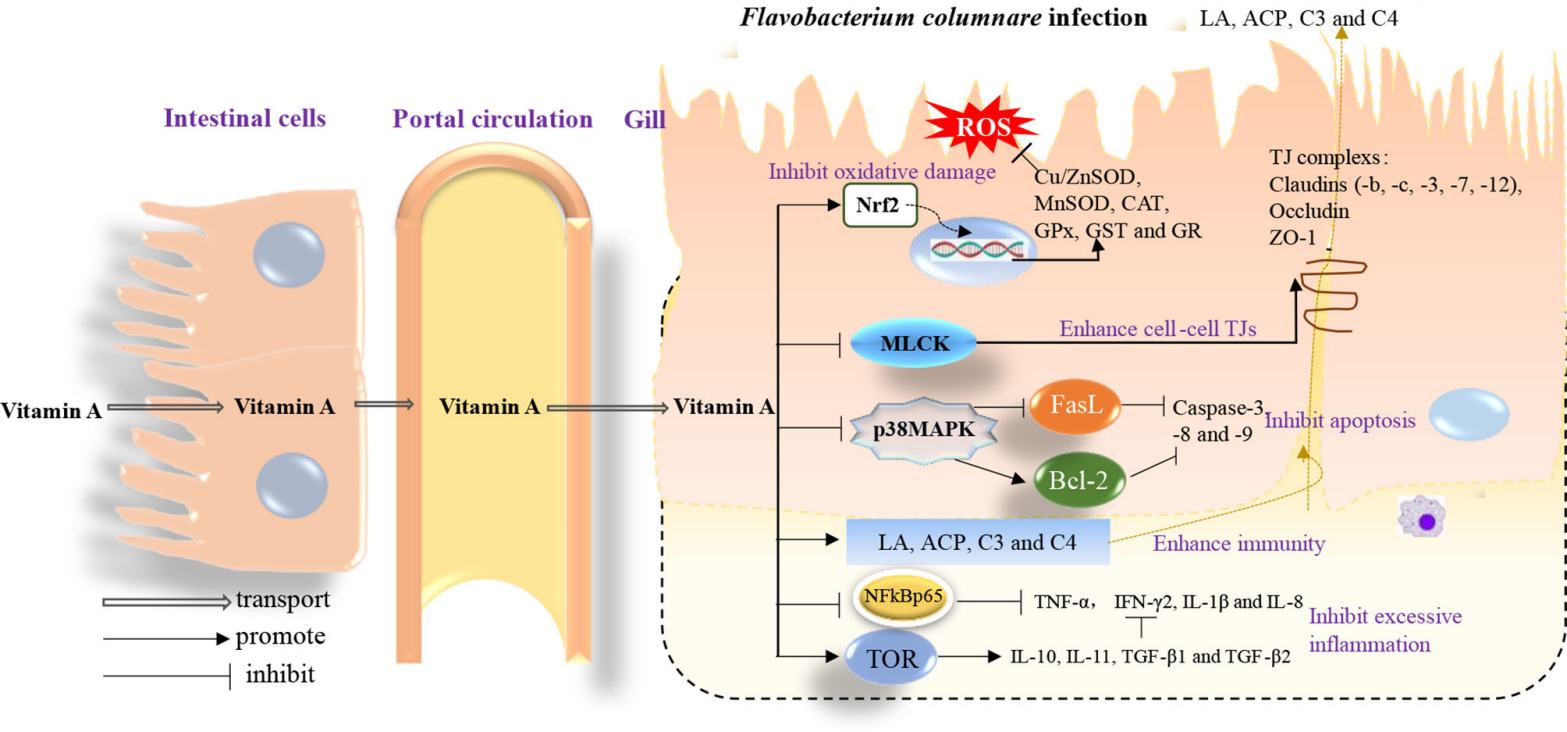
A schematic diagram representing the mode of action of vitamin A on the immune response and structural integrity of fish gills.

Here, we have elegantly demonstrated for the first time that vitamin A deficiency can effectively damage the resistance of fish gill rot possibly through impairing fish gill immune and physical barrier function, whereas optimal vitamin A can reverse these negative effects (**Figure 8**). Firstly, vitamin A significantly enhanced fish gill immune function, which might be related to promote fish gill antibacterial ability, and in part might be related to the inhibition of (IKKβ, IKKγ)/IκBα/NF-κB p65-mediated NF-κB canonical signaling pathway (rather than IKKα/NF-κB p52-mediated non-canonical signaling pathway) and stimulation of activation of TOR/S6K1 signaling pathways to mitigate an inflammatory response in fish gill. Secondly, dietary vitamin A could substantially enhance gill physical barrier that might be related to 1) enhancement in fish gill antioxidant capacity, which might be partly by an increase of non-enzymatic antioxidant components GSH and enzymatic antioxidant system associated with the promotion of Nrf2 signaling pathway referring to downregulating *keap1a* and *keap1b* mRNA levels; 2) inhibition of fish gill cell apoptosis, which can occur due to downregulation of both *caspase-8* and *caspase-9* mRNA levels, thus resulting in a reduction of caspase-3 (rather than *caspase-7*) mRNA levels concomitant with the inhibition of p38MAPK signaling pathway; 3) maintenance of fish gill cell-to-cell integrity partly by upregulating TJ mRNA levels (excluding that of *claudin-15*) related to the inhibition of MLCK signaling pathway. Furthermore, based on protection conferred on fish against the gill rot morbidity, ACP activity, and against the lipid peroxidation (MDA content), the optimum vitamin A levels for on-growing grass carp were found to be 1,991, 2,188, and 2,934 IU/kg diet, respectively.

## Data Availability Statement

The original contributions presented in the study are included in the article/**Supplementary Material**. Further inquiries can be directed to the corresponding author.

## Ethics Statement

The animal study was reviewed and approved by The Animal Care and Use Committee of Sichuan Agricultural University. Written informed consent was obtained from the owners for the participation of their animals in this study.

## Author Contributions

WJ: conceptualization, methodology, validation and resources, writing—review and editing. LiZ: investigation and data curation, writing—original draft. LF: conceptualization, formal analysis. PW: methodology. YL: writing—review and editing. SK: validation and resources. SL: validation and resources. LT: validation and resources. HM: analyzed and interpreted the data. LuZ: analyzed and interpreted the data. XZ: funding acquisition, project administration and supervision.

## Funding

This research was financially supported by the National Natural Science Foundation of China (31972810), National Key R&D Program of China (2019YFD0900200 and 2018YFD0900400), Outstanding Youth Science Foundation of Sichuan Province (2020JDJQ0043), National Natural Science Foundation of China for Outstanding Youth Science Foundation (31922086), China Agriculture Research System of MOF and MARA (CARS-45), and Sichuan Science and Technology Program (2019YFN0036). The authors would like to thank the personnel of these teams for their kind assistance.

## Conflict of Interest

S-YK, S-WL, and LT are employed by Sichuan Animtech Feed Co., Ltd. H-FM and LZ are employed by Tongwei Co., Ltd.

The remaining authors declare that the research was conducted in the absence of any commercial or financial relationships that could be construed as a potential conflict of interest.

## Publisher’s Note

All claims expressed in this article are solely those of the authors and do not necessarily represent those of their affiliated organizations, or those of the publisher, the editors and the reviewers. Any product that may be evaluated in this article, or claim that may be made by its manufacturer, is not guaranteed or endorsed by the publisher.
