# Supplementary material for: New Insight on the Immune Modulation and Physical Barrier Protection Caused by Vitamin A in Fish Gills Infected With Flavobacterium columnare
Source: Front Immunol. 2022 Mar 25;13:833455. doi: 10.3389/fimmu.2022.833455 (PMC8992971; doi:10.3389/fimmu.2022.833455)
Supplement: Supplementary file 1 [file DataSheet_1.docx]

**Table S1** Formulation and nutrient content of the basal diet

| **Ingredients** | **%** | **Nutrients content %** | |
| --- | --- | --- | --- |
| **Fish meal** | 15.55 | **Crude protein^4^** | 29.71 |
| **Soybean protein concentrate** | 26.25 | **Crude lipid^4^** | 3.58 |
| **Gelatin** | 3.13 | **n-3^5^** | 0.50 |
| **α-starch** | 24.00 | **n-6^5^** | 1.00 |
| **Corn starch** | 16.323 | **Available phosphorus^5^** | 0.84 |
| **Soybean oil** | 1.931 |  |  |
| **Cellulose** | 5.00 |  |  |
| **L-met (98%)** | 0.401 |  |  |
| **Ca(H_2_PO_4_)_2_** | 2.865 |  |  |
| **Vitamin premix^1^** | 1.00 |  |  |
| **Mineral premix^2^** | 2.00 |  |  |
| **Vitamin A premix^3^** | 1.00 |  |  |
| **Choline chloride (60%)** | 0.50 |  |  |
| **Ethoxyquin (30%)** | 0.05 |  |  |

^1^ Per kilogram of vitamin premix (g/kg): cholecalciferol (500,000IU/g), 0.40; D, L-α-tocopherol acetate (50%), 12.58; menadione (22.9%), 0.83; cyanocobalamin (1%), 0.94; D-biotin (2%), 0.75; folic acid (95%), 0.42; thiamine nitrate (98%), 0.11; ascorhyl acetate (95%), 4.31; niacin (99%), 2.58; meso-inositol (98%), 19.39; calcium-D-pantothenate (98%), 2.56; riboflavin (80%), 0.63; pyridoxine hydrochloride (98%), 0.62. All ingredients were diluted with corn starch to 1 kg.

^2^ Per kilogram of mineral premix (g/kg): MnSO_4_.H_2_O (31.8% Mn), 1.8900; MgSO_4_⋅H_2_O (15.0% Mg), 200.0000; FeSO_4_.H_2_O (30.0% Fe), 24.5700; ZnSO_4_.H_2_O (34.5% Zn), 8.2500; CuSO_4_.5H_2_O (25.0% Cu), 0.9600; KI (76.9% I), 0.0668g; Na_2_SeO_3_ (44.7% Se), 0.0168. All ingredients were diluted with corn starch to 1 kg.

^3^ Vitamin A premix: premix was added to obtain graded level of vitamin A and the amount of corn starch was reduced to compensate.

^4^ Crude protein and crude lipid contents were measured value.

^5^ Available phosphorus, n-3 and n-6 contents were calculated according to NRC (2011).

**Table S2** Real-time PCR information

| Genes | Primer sequence  Forward (5^’^→3^’^) | Primer sequence  Reverse (5^’^→3^’^) | Thermocycling conditions | Accession number |
| --- | --- | --- | --- | --- |
| β-defensin | TTGCTTGTCCTTGCCGTCT | AATCCTTTGCCACAGCCTAA | 95 °C 30 s, 40 cycles of 95 °C 5 s, 58.4 °C 30 s and 72 °C 30 s | KT445868 |
| hepcidin | AGCAGGAGCAGGATGAGC | GCCAGGGGATTTGTTTGT | 95 °C 30 s, 40 cycles of 95 °C 5 s, 59.3 °C 30 s and 72 °C 30 s | JQ246442.1 |
| LEAP-2A | TGCCTACTGCCAGAACCA | AATCGGTTGGCTGTAGGA | 95 °C 30 s, 40 cycles of 95 °C 5 s, 59.3 °C 30 s and 72 °C 30 s | FJ390414 |
| LEAP-2B | TGTGCCATTAGCGACTTCTGAG | ATGATTCGCCACAAAGGGG | 95 °C 30 s, 40 cycles of 95 °C 5 s, 59.3 °C 30 s and 72 °C 30 s | KT625603 |
| TNF-α | CGCTGCTGTCTGCTTCAC | CCTGGTCCTGGTTCACTC | 95 °C 30 s, 40 cycles of 95 °C 5 s, 58.4 °C 30 s and 72 °C 30 s | HQ696609 |
| IFN-γ2 | TGTTTGATGACTTTGGGATG | TCAGGACCCGCAGGAAGAC | 95 °C 30 s, 40 cycles of 95 °C 5 s, 60.4 °C 30 s and 72 °C 30 s | JX657682 |
| IL-1β | AGAGTTTGGTGAAGAAGAGG | TTATTGTGGTTACGCTGGA | 95 °C 30 s, 40 cycles of 95 °C 5 s, 57.1 °C 30 s and 72 °C 30 s | JQ692172 |
| IL-8 | ATGAGTCTTAGAGGTCTGGGT | ACAGTGAGGGCTAGGAGGG | 95 °C 30 s, 40 cycles of 95 °C 5 s, 60.3 °C 30 s and 72 °C 30 s | JN663841 |
| IL-10 | AATCCCTTTGATTTTGCC | GTGCCTTATCCTACAGTATGTG | 95 °C 30 s, 40 cycles of 95 °C 5 s, 61.4 °C 30 s and 72 °C 30 s | HQ388294 |
| IL-11 | GGTTCAAGTCTCTTCCAGCGAT | TGCGTGTTATTTTGTTCAGCCA | 95 °C 30 s, 40 cycles of 95 °C 5 s, 57.0 °C 30 s and 72 °C 30 s | KT445870 |
| TGF-β1 | TTGGGACTTGTGCTCTAT | AGTTCTGCTGGGATGTTT | 95 °C 30 s, 40 cycles of 95 °C 5 s, 55.9 °C 30 s and 72 °C 30 s | EU099588 |
| TGF-β2 | TACATTGACAGCAAGGTGGTG | TCTTGTTGGGGATGATGTAGTT | 95 °C 30 s, 40 cycles of 95 °C 5 s, 55.9 °C 30 s and 72 °C 30 s | KM279716 |
| NF-κB p52 | TCAGTGTAACGACAACGGGAT | ATACTTCAGCCACACCTCTCTTAG | 95 °C 30 s, 40 cycles of 95 °C 5 s, 58.4 °C 30 s and 72 °C 30 s | KM279720 |
| NF-κB p65 | GAAGAAGGATGTGGGAGATG | TGTTGTCGTAGATGGGCTGAG | 95 °C 30 s, 40 cycles of 95 °C 5 s, 62.3 °C 30 s and 72 °C 30 s | KJ526214 |
| IκBα | TCTTGCCATTATTCACGAGG | TGTTACCACAGTCATCCACCA | 95 °C 30 s, 40 cycles of 95 °C 5 s, 62.3 °C 30 s and 72 °C 30 s | KJ125069 |
| IKKα | GGCTACGCCAAAGACCTG | CGGACCTCGCCATTCATA | 95 °C 30 s, 40 cycles of 95 °C 5 s, 60.3 °C 30 s and 72 °C 30 s | KM279718 |
| IKKβ | GTGGCGGTGGATTATTGG | GCACGGGTTGCCAGTTTG | 95 °C 30 s, 40 cycles of 95 °C 5 s, 60.3 °C 30 s and 72 °C 30 s | KP125491 |
| IKKγ | AGAGGCTCGTCATAGTGG | CTGTGATTGGCTTGCTTT | 95 °C 30 s, 40 cycles of 95 °C 5 s, 58.4 °C 30 s and 72 °C 30 s | KM079079 |
| TOR | TCCCACTTTCCACCAACT | ACACCTCCACCTTCTCCA | 95 °C 30 s, 40 cycles of 95 °C 5 s, 61.4 °C 30 s and 72 °C 30 s | JX854449 |
| S6K1 | TGGAGGAGGTAATGGACG | ACATAAAGCAGCCTGACG | 95 °C 30 s, 40 cycles of 95 °C 5 s, 54.0 °C 30 s and 72 °C 30 s | EF373673 |
| 4E-BP1 | GCTGGCTGAGTTTGTGGTTG | CGAGTCGTGCTAAAAAGGGTC | 95 °C 30 s, 40 cycles of 95 °C 5 s, 60.3 °C 30 s and 72 °C 30 s | KT757305 |
| 4E-BP2 | CACTTTATTCTCCACCACCCC | TTCATTGAGGATGTTCTTGCC | 95 °C 30 s, 40 cycles of 95 °C 5 s, 60.3 °C 30 s and 72 °C 30 s | KT757306 |
| caspase-3 | GCTGTGCTTCATTTGTTTG | TCTGAGATGTTATGGCTGTC | 95 °C 30 s, 40 cycles of 95 °C 5 s, 55.9 °C 30 s and 72 °C 30 s | JQ793789 |
| caspase-7 | GCCATTACAGGATTGTTTCACC | CCTTATCTGTGCCATTGCGT | 95 °C 30 s, 40 cycles of 95 °C 5 s, 57.1 °C 30 s and 72 °C 30 s | KT625601 |
| caspase-8 | ATCTGGTTGAAATCCGTGAA | TCCATCTGATGCCCATACAC | 95 °C 30 s, 40 cycles of 95 °C 5 s, 59.0 °C 30 s and 72 °C 30 s | KM016991 |
| caspase-9 | CTGTGGCGGAGGTGAGAA | GTGCTGGAGGACATGGGAAT | 95 °C 30 s, 40 cycles of 95 °C 5 s, 59.0 °C 30 s and 72 °C 30 s | JQ793787 |
| Apaf-1 | AAGTTCTGGAGCCTGGACAC | AACTCAAGACCCCACAGCAC | 95 °C 30 s, 40 cycles of 95 °C 5 s, 61.4 °C 30 s and 72 °C 30 s | KM279717 |
| Bax | CATCTATGAGCGGGTTCGTC | TTTATGGCTGGGGTCACACA | 95 °C 30 s, 40 cycles of 95 °C 5 s, 60.3 °C 30 s and 72 °C 30 s | JQ793788.1 |
| FasL | AGGAAATGCCCGCACAAATG | AACCGCTTTCATTGACCTGGAG | 95 °C 30 s, 40 cycles of 95 °C 5 s, 61.4 °C 30 s and 72 °C 30 s | KT445873 |
| Bcl-2 | AGGAAAATGGAGGTTGGGAT | CTGAGCAAAAAAGGCGATG | 95 °C 30 s, 40 cycles of 95 °C 5 s, 60.3 °C 30 s and 72 °C 30 s | JQ713862.1 |
| p38MAPK | TGGGAGCAGACCTCAACAAT | TACCATCGGGTGGCAACATA | 95 °C 30 s, 40 cycles of 95 °C 5 s, 60.4 °C 30 s and 72 °C 30 s | KM112098 |
| Cu/ZnSOD | CGCACTTCAACCCTTACA | ACTTTCCTCATTGCCTCC | 95 °C 30 s, 40 cycles of 95 °C 5 s, 61.5 °C 30 s and 72 °C 30 s | GU901214 |
| MnSOD | ACGACCCAAGTCTCCCTA | ACCCTGTGGTTCTCCTCC | 95 °C 30 s, 40 cycles of 95 °C 5 s, 60.4 °C 30 s and 72 °C 30 s | GU218534 |
| CAT | GAAGTTCTACACCGATGAGG | CCAGAAATCCCAAACCAT | 95 °C 30 s, 40 cycles of 95 °C 5 s, 58.7 °C 30 s and 72 °C 30 s | FJ560431 |
| GPx1a | GGGCTGGTTATTCTGGGC | AGGCGATGTCATTCCTGTTC | 95 °C 30 s, 40 cycles of 95 °C 5 s, 61.5 °C 30 s and 72 °C 30 s | EU828796 |
| GPx1b | TTTTGTCCTTGAAGTATGTCCGTC | GGGTCGTTCATAAAGGGCATT | 95 °C 30 s, 40 cycles of 95 °C 5 s, 60.3 °C 30 s and 72 °C 30 s | KT757315 |
| GPx4a | TACGCTGAGAGAGGTTTACACAT | CTTTTCCATTGGGTTGTTCC | 95 °C 30 s, 40 cycles of 95 °C 5 s, 60.4 °C 30 s and 72 °C 30 s | KU255598 |
| GPx4b | CTGGAGAAATACAGGGGTTACG | CTCCTGCTTTCCGAACTGGT | 95 °C 30 s, 40 cycles of 95 °C 5 s, 60.3 °C 30 s and 72 °C 30 s | KU255599 |
| GSTr | TCTCAAGGAACCCGTCTG | CCAAGTATCCGTCCCACA | 95 °C 30 s, 40 cycles of 95 °C 5 s, 58.4 °C 30 s and 72 °C 30 s | EU107283 |
| GR | GTGTCCAACTTCTCCTGTG | ACTCTGGGGTCCAAAACG | 95 °C 30 s, 40 cycles of 95 °C 5 s, 59.4 °C 30 s and 72 °C 30 s | JX854448 |
| Nrf2 | CTGGACGAGGAGACTGGA | ATCTGTGGTAGGTGGAAC | 95 °C 30 s, 40 cycles of 95 °C 5 s, 62.5 °C 30 s and 72 °C 30 s | KF733814 |
| keap1a | TTCCACGCCCTCCTCAA | TGTACCCTCCCGCTATG | 95 °C 30 s, 40 cycles of 95 °C 5 s, 63.0 °C 30 s and 72 °C 30 s | KF811013 |
| keap1b | TCTGCTGTATGCGGTGGGC | CTCCTCCATTCATCTTTCTCG | 95 °C 30 s, 40 cycles of 95 °C 5 s, 57.9 °C 30 s and 72 °C 30 s | KJ729125 |
| claudin-b | GAGGGAATCTGGATGAGC | ATGGCAATGATGGTGAGA | 95 °C 30 s, 40 cycles of 95 °C 5 s, 57.0 °C 30 s and 72 °C 30 s | KF193860 |
| claudin-c | GAGGGAATCTGGATGAGC | CTGTTATGAAAGCGGCAC | 95 °C 30 s, 40 cycles of 95 °C 5 s, 59.4 °C 30 s and 72 °C 30 s | KF193859 |
| claudin-3 | ATCACTCGGGACTTCTA | CAGCAAACCCAATGTAG | 95 °C 30 s, 40 cycles of 95 °C 5 s, 57.0 °C 30 s and 72 °C 30 s | KF193858 |
| claudin-7 | ACTTACCAGGGACTGTGGATGT | CACTATCATCAAAGCACGGGT-3 | 95 °C 30 s, 40 cycles of 95 °C 5 s, 59.3 °C 30 s and 72 °C 30 s | KT625604 |
| claudin-12 | CCCTGAAGTGCCCACAA | GCGTATGTCACGGGAGAA | 95 °C 30 s, 40 cycles of 95 °C 5 s, 55.4 °C 30 s and 72 °C 30 s | KF998571 |
| claudin-15a | TGCTTTATTTCTTGGCTTTC | CTCGTACAGGGTTGAGGTG | 95 °C 30 s, 40 cycles of 95 °C 5 s, 59.0 °C 30 s and 72 °C 30 s | KF193857 |
| occludin | TATCTGTATCACTACTGCGTCG | CATTCACCCAATCCTCCA | 95 °C 30 s, 40 cycles of 95 °C 5 s, 59.4 °C 30 s and 72 °C 30 s | KF193855 |
| ZO-1 | CGGTGTCTTCGTAGTCGG | CAGTTGGTTTGGGTTTCAG | 95 °C 30 s, 40 cycles of 95 °C 5 s, 59.4 °C 30 s and 72 °C 30 s | KJ000055 |
| MLCK | GAAGGTCAGGGCATCTCA | GGGTCGGGCTTATCTACT | 95 °C 30 s, 40 cycles of 95 °C 5 s, 53.0 °C 30 s and 72 °C 30 s | KM279719 |
| β-actin | GGCTGTGCTGTCCCTGTA | GGGCATAACCCTCGTAGAT | 95 °C 30 s, 40 cycles of 95 °C 5 s, 61.4 °C 30 s and 72 °C 30 s | M25013 |

LEAP-2, liver expressed antimicrobial peptide 2; IFN-γ2, interferonγ2; TNF-a, tumor necrosis factor a; IL, interleukin; TGF-β, transforming growth factor β; NF-κB, nuclear factor kappa B; IκBa, inhibitor of kBa; IKK, IκB kinase; TOR, target of rapamycin; S6K1, ribosome protein S6 kinase 1; 4E-BP1, eukaryotic translation initiation factor 4E-binding protein 1; Apaf-1, apoptotic protease activating factor 1; Bax, B-cell lymphoma protein 2 associated X protein; FasL, Fas ligand; Bcl-2, B-cell leukaemia/lymphoma-2; p38MAPK, p38 mitogen-activated protein kinase; Cu/ZnSOD, copper/zinc superoxide dismutase; MnSOD, manganese superoxide dismutase; CAT, catalase; GPx, glutathione peroxidase; GST, glutathione-*S*-transferase; GR, glutathione reductase; Nrf2, NF-E2-related factor 2; keap1, kelch-like-ECH-associated protein 1; ZO-1, zonula occludens 1; MLCK, myosin light chain kinase

**Table S3** Correlation coefﬁcients

| Dependent parameters | Independent parameters | Correlation coefﬁcients | *P* |
| --- | --- | --- | --- |
| TNF-α | NF-κB P65 | +0.995 | < 0.01 |
| IFN-γ2 | NF-κB P65 | +0.897 | < 0.05 |
| IL-1β | TOR | – 0.948 | < 0.01 |
|  | S6K1 | – 0.899 | < 0.05 |
| IL-8 | NF-κB P65 | +0.934 | < 0.01 |
| IL-10 | NF-κB P65 | -0.880 | < 0.05 |
| IL-11 | TOR | +0.887 | < 0.05 |
|  | S6K1 | +0.883 | < 0.05 |
| TGF-β1 | TOR | +0.928 | < 0.01 |
|  | S6K1 | +0.855 | < 0.05 |
| TGF-β2 | NF-κB P65 | – 0.950 | < 0.01 |
| NF-κB P65 | IκBα | – 0.933 | < 0.01 |
| IκBα | IKKβ | – 0.896 | < 0.05 |
|  | IKKγ | – 0.982 | < 0.01 |
| caspase-3 | caspase-8 | +0.948 | < 0.01 |
|  | caspase-9 | +0.915 | < 0.05 |
| caspase-8 | FasL | +0.839 | < 0.05 |
|  | P38MAPK | +0.961 | < 0.01 |
| caspase-9 | Apaf-1 | +0.929 | < 0.01 |
|  | Bax | +0.899 | < 0.05 |
|  | P38MAPK | +0.884 | < 0.05 |
| MnSOD activity | MnSOD mRNA | +0.889 | < 0.05 |
| GPx activity | GPx4b mRNA | +0.935 | < 0.01 |
| GST activity | GSTr mRNA | +0.965 | < 0.01 |
| GR activity | GR mRNA | +0.883 | < 0.05 |
| Nrf2 | CuZnSOD  MnSOD | +0.945  +0.951 | < 0.01  < 0.01 |
|  | GPx4a | +0.917 | < 0.01 |
|  | GPx4b | +0.971 | < 0.01 |
|  | GSTr | +0.903 | < 0.05 |
|  | GR | +0.818 | < 0.05 |
| Keap1a | Nrf2 | – 0.934 | < 0.01 |
| MLCK | Claudin-b  Claudin-c | – 0.920  – 0.846 | < 0.01  < 0.05 |
|  | Claudin-3  Occludin | – 0.892  – 0.953 | < 0.05  < 0.01 |
|  | ZO-1 | – 0.950 | < 0.01 |
